# Outcome Analysis of Total Thyroidectomy: Conventional Suture Ligation Technique vs Sutureless Technique

**DOI:** 10.7759/cureus.48005

**Published:** 2023-10-30

**Authors:** Sebastian Jesu Thayalan Dias, Sreekanthan Gobishangar, Kuganthan Priyatharsan, Shathana Praramanathan

**Affiliations:** 1 Department of Surgery, Jaffna Teaching Hospital, Jaffna, LKA; 2 Department of Surgery, Faculty of Medicine, University of Jaffna, Jafffna, LKA; 3 Department of Surgery, University of Jaffna, Jaffna, LKA; 4 Department of Health Sciences, Management and Science University, Shah Alam, MYS

**Keywords:** endocrine surgery, operative time, conventional suture ligation, sutureless technique, total thyroidectomy

## Abstract

Introduction: Total thyroidectomy is a common surgical procedure in endocrine surgery. However, it carries potential complications such as damage to the recurrent laryngeal nerve, permanent hypoparathyroidism, and bleeding.

Methods: A prospective study was conducted at the Professorial Surgical Unit, Jaffna Teaching Hospital, involving consecutive patients who underwent total thyroidectomy. Patients with certain conditions were excluded from the study. The data collected were analyzed using IBM SPSS Statistics for Windows, Version 25 (Released 2017; IBM Corp., Armonk, New York, United States).

Results: This study included 59 patients who had total thyroidectomy from January 2018 to January 2021 at the Professorial Surgical Unit, Jaffna Teaching Hospital. Of these, 45 underwent conventional suture ligation (CSL), and 17 had a sutureless (SL) technique. Mean ages were 44±12.47 years (range: 23 to 68) for CSL and 47.63±13.37 years (range: 27 to 73) for SL. Operative time was 2.16 ± 0.32 hours for CSL and 1.56 ± 0.49 hours for SL. Intraoperative and postoperative bleeding occurred in 2.38% of CSL cases but not in SL. Postoperative hypocalcemia was 7.14% for CSL and 5.88% for SL. Postoperative stays averaged 3.83 ± 1.56 days for CSL and 3.41 ± 1.62 days for SL.

Discussion: The study found that the operative time differed significantly between the suture and SL techniques. However, there was no statistically significant difference in postoperative drainage volume or postoperative complications.

Conclusion: The SL technique was shown to be superior to the conventional suture ligation technique for total thyroidectomy. It resulted in shorter operative time, reduced intraoperative bleeding, lower incidence of postoperative drainage, fewer postoperative voice changes, and shorter hospital stays. Therefore, the SL technique was deemed safe, efficient, and effective for total thyroidectomy compared to the conventional suture ligation technique.

## Introduction

Total thyroidectomy is one of the commonest surgical procedures in endocrine surgery [1-14]. It is performed for various indications, including malignancies and benign thyroid swellings. Even though it is a common surgery, there are identified complications like any other surgical intervention. A few important complications include damage to the recurrent laryngeal nerve, permanent hypoparathyroidism (due to accidental removal of all parathyroid glands or damaging its blood supply), and intraoperative and postoperative bleeding [2-4]. Ensuring effective hemostasis is of utmost importance for thyroid surgeons in order to prevent potential complications [14]. Besides the aforementioned priority, streamlining operative time has become an increasingly important consideration [15]. Hemostasis can be achieved using conventional suture ligation (CSL) and the sutureless (SL) technique. This study compares the surgical results between the conventional suture ligation technique and sutureless vascular technique by using bipolar diathermy/ harmonic scalpel /ligature device in terms of recurrent laryngeal nerve (RLN) damage, duration of surgery, postoperative bleeding, the incidence of hypoparathyroidism, and duration of the hospital stay.

## Materials and methods

A prospective cohort study was conducted at the Professorial Surgical Unit, Jaffna Teaching Hospital, from January 2018 to January 2021. Ethical approval was obtained from the Ethics Review Committee of the Faculty of Medicine, University of Jaffna (J/ERC/21/122/NDR/0241). The study was conducted in all consecutive patients who underwent total thyroidectomy, excluding patients with extra thyroid invasion of thyroid malignancy and radiation treatment history to the thyroid gland or radioiodine ablation. The selected patients of both the SL technique and CSL technique were observed in the ward and asked to follow up in the surgical clinic to maintain the postoperative records for the secondary and delayed complications.

Preoperative patients’ details and clinical evaluation, including the biochemical parameters, were obtained, including preoperative voice change, vocal cord palsy, stridor, and preoperative serum calcium. Intraoperative information such as the duration of the surgery, quantity of the intraoperative bleeding, and postoperative clinical and biochemical assessment such as temporary RLN damage, hypocalcemia (<1.12mmol/L), transient or temporary hypoparathyroidism, drainage volume, and signs of infection was also obtained. The postoperative evaluation comprised an overall analysis of biochemical parameters in hypocalcemia, voice change in permanent RLN damage, and histopathology reports for encountered malignancies.

Collected data were analyzed, processed, and saved on the personal computer of the investigators. Analysis was done based on research problems, objectives, and types of variables. The continuous variables were reported as the mean and standard deviation (SD), and the categorical variables were tabulated as the absolute number and a percentage of the total. Statistical analysis was done using IBM SPSS Statistics for Windows, Version 25 (Released 2017; IBM Corp., Armonk, New York, United States) using the two-way Student t-test for continuous variables and the Chi-squared test for categorical equations. The p-value less than 0.05 was considered statistically significant.

## Results

**Figure 1:**
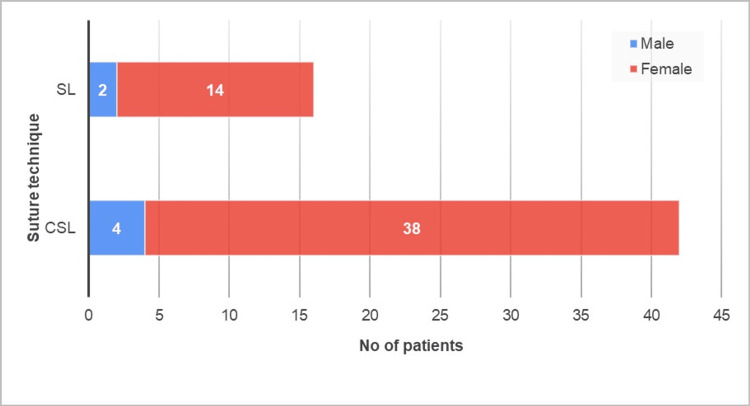
Distribution of the suture technique among gender SL: Sutureless; CSL: conventional suture ligation

This study included 59 patients undergoing total thyroidectomy from January 2018 to January 2021 in the Professorial Surgical unit, Teaching Hospital, Jaffna. They are categorized as 45 patients for the CSL technique and 17 for the SL technique. Their age as a mean is 44±12.47 (range 23 to 68) in CSL and 47.63±13.37 (range 27 to 73) in SL. The male-to-female ratio was 1:9.5 and 1:7.5 in CSL and SL, respectively (Figure 1). The baseline characteristics were compared between the two techniques, CSL and SL (Table 1). Table 1 summarizes the perioperative results of the CSL and SL technique.

**Table 1:** Summarized perioperative results of the conventional suture ligation and sutureless technique

Parameters		Suture	Sutureless
Number of patients		42	17
Age (Mean and SD)		44.5 ± 12.47	47.63 ± 13.37
Sex (Male: female ratio)		1 : 9.5	1 : 7.5
Preop voice change		2.38%	5.9%
Preop vocal cord		0%	0%
Preop serum calcium (hypocalcemia)		7.14%	5.88%
Preop stridor		0%	0%
Operative time		2.16 ± 0.32 h	1.56 ± 0.49 h
Intraoperative bleeding (significant)		2.38%	0%
Postop voice change		2.38%	0%
Postop vocal cord		0%	0%
Drain	Not used	11.9%	81.25%
	Used	88.1%	18.75%
Drain volume		56.29 ± 38.70 cc	50.57 ± 27.74 cc
Postop Day 3 serum calcium (Hypocalcemia)		7.14%	5.88%
Postop hospital stay		3.83 ± 1.56 days	3.41 ± 1.62 days

In the preoperative assessment, it was observed that 2.38% of patients in the suture group experienced voice changes, whereas a slightly higher incidence of 5.9% was noted in the SL group. Notably, none of the patients in either group exhibited preoperative vocal cord issues. Additionally, a comparison of serum calcium levels revealed that 7.14% of patients in the suture group had hypocalcemia, defined as low serum calcium levels, while a slightly lower prevalence of 5.88% was observed in the SL group. Importantly, preoperative stridor, a potentially distressing symptom, was absent in both study groups.

During the operative phase of the study, significant disparities emerged between the suture and SL groups. Notably, the mean operative time for the suture group was found to be 2.16 hours (SD = 0.32), which was significantly longer than the mean operative time of 1.56 hours (SD = 0.49) in the SL group. This divergence in operative durations highlights a substantial difference in the efficiency of the two techniques.

Furthermore, intraoperative bleeding during surgical procedures was assessed. In this regard, 2.38% of patients in the suture group experienced significant bleeding during surgery. In contrast, none of the patients in the SL group encountered significant intraoperative bleeding.

Postoperative voice changes were reported in 2.38% of patients who underwent the suture technique, highlighting a minor but notable issue. Conversely, no voice changes were observed in the SL group. Notably, neither group experienced postoperative vocal cord issues.

The use of surgical drains differed substantially between the two groups. In the SL group, 11.9% of patients did not require a drain, while a substantial majority of 88.1% of suture patients necessitated drain placement because we didn't expect significant oozing from the dissected surface. Moreover, the mean drain volume was slightly lower in the SL group, with 50.57 cc (SD = 27.74), compared to 56.29 cc (SD = 38.70) in the suture group. 

Postoperative hypocalcemia on Day 3 was noted in 7.14% of patients who underwent the suture technique, while a slightly lower incidence of 5.88% was observed in the SL group. Although both groups experienced some cases of hypocalcemia, this parameter did not demonstrate a marked difference between the techniques.

Finally, the mean postoperative hospital stay in the suture group was 3.83 days (SD = 1.56), while in the SL group, it was 3.41 days (SD = 1.62). These results indicate a comparable postoperative recovery duration for both techniques.

## Discussion

Total thyroidectomy is one of the most common endocrine surgeries in the world [1]. Since the evolution of bloodless surgeries, total thyroidectomy has also had many advances. Several studies were conducted in various parts of the world to compare CSL and SL techniques. Studies have suggested the superiority of SL over CSL in various aspects of intraoperative and postoperative outcomes [5-8].

The mean age for the suture method was 44.5 ± 12.47 years, while for the SL method, it was 47.63 ± 13.37 years. In another study, the mean age for SL thyroidectomy was 39.85 ± 8.47 years, and for the conventional group, it was 43.17 ± 9.69 years [12].

Postoperative drainage volume in the suture technique was 56.29 ± 38.70 cc while it was 50.57 ± 27.74 cc in the SL technique. Most of the dissection is near the gland/ capsule of the thyroid we did not expect significant drain in these patients. A significant difference was observed between the postoperative blood volume in the study conducted in Iraq (p=0.046 and 95%) [5]. A study showed that the first 24 h postoperative drainage volumes of the conventional technique group had the mean ± SD 48.18 ± 12.10; this is much lower than that in the present study as the drain was collected for the first 24 hours. Statistically, there was no significant difference observed in the study (p-value = 0.447) [9]

In our study, it was observed that the postoperative hospital stay of the CSL technique is 3.83 ± 1.56 days, while the SL technique showed 3.41 ± 1.62 days. The p-value is 0.152 and statistically not significant. The hospital stay for CSL was comparatively much shorter in the studies in Iraq and Turkey (2.7 ± 0.6 days, 1.7 ± 0.60 days) and almost equal in the study done in Pakistan (3.7 ± 1.3 days) [10]. Studies in Iraq and Turkey also showed p-values of 0.298 and 0.268 in their studies, which were statistically not significant [5,9].

The operative time in our study was 2.16 ± 0.32 h for the suture technique and 1.56 ± 0.49 h for the SL technique and the p-value is 0.04 which is statistically significant. The operative time taken for the conventional suture method was observed to be longer than the SL technique in this study. In the study in Iraq, operative time (1.88 ± 0.18 hour) was taken for the conventional suture method, which is comparatively lower than our study and the study showed statistical significance, p<0.001 between conventional suture technique and SL technique. In the study conducted in Australia among 1945 patients who underwent total thyroidectomy, the author observed that the mean of operative time was significantly lower in the SL group than in the conventional method group (1.43 hours and SL technique 1.18 hours) [11]. In a study, the time taken for the conventional method operation was 2.68 ± 0.70 hours, whereas the SL technique took 2.20 ± 0.65h and showed statistical significance between the conventional method and SL method (harmonic scalpel group) [7].

In this prospective cohort study of 59 subjects, we have found that postoperative need for a drain (drain used in CSL 88.1% and SL 18.75%) and postoperative voice changes (in CSL 2.38% and SL 0%) are much less in the SL technique than conventional suture ligation technique using the bipolar diathermy device, harmonic scalpel, and ligature device available in Teaching Hospital Jaffna collectively.

Hypoparathyroidism will manifest biochemically as hypocalcemia, an important postoperative complication of total thyroidectomy. It can occur temporarily as well as permanently. Hypocalcemia may appear due to direct trauma, accidental removal, ischemic injury, or over-handling of the parathyroid gland. There was no significant difference in our study. This study noted the preoperative calcium level and calcium level on postop day 3 and it was noted that hypocalcemia was noted in 7.14% in CSL and 5.88% in SL and a total of 13.02% of patients had hypocalcemia. A study done in Iraq showed that an equal number of patients had hypocalcemia in the CSL and SL groups (11.25%) [5]. However, the Iran study assessed the serum calcium by noting the serum calcium levels on 1st, 2nd 10th, and 30th days postoperatively and assessed the transient and permanent hypocalcemia [5].

Limitation

The study's sample size was constrained due to the single-center nature of the investigation and data collection during the COVID-19 pandemic. This limitation may restrict the generalizability of the findings to a wider surgical context. Additionally, the reduced surgical caseload during the pandemic may have introduced a bias in the types of cases included in the study.

## Conclusions

Regarding total thyroidectomy, the SL technique was superior to the conventional suture ligation technique as the former had shorter operative time, reduced amount of intraoperative bleeding, low incidence of the postoperative drain, decreased postoperative changes of voice, and a smaller number of days of hospital stay following the surgery. Total thyroidectomy is a surgical procedure done in a relatively highly vascular field. Even though postoperative bleeding is rare, it is a potentially life-threatening complication. Taking this into account, the SL technique is a safe, efficient, and effective method of total thyroidectomy compared to the CSL technique.
